# Investigations of Electronic, Structural, and In Silico Anticancer Potential of Persuasive Phytoestrogenic Isoflavene-Based Mannich Bases

**DOI:** 10.3390/molecules28155911

**Published:** 2023-08-06

**Authors:** Sadaf Mutahir, Muhammad Asim Khan, Maryam Mushtaq, Haishan Deng, Ahmed M. Naglah, Abdulrahman A. Almehizia, Mohamed A. Al-Omar, Faris Ibrahim Alrayes, Atef Kalmouch, Shaima A. El-Mowafi, Moamen S. Refat

**Affiliations:** 1 School of Chemistry and Chemical Engineering, Linyi University, Linyi 276000, China; 2 Department of Chemistry, University of Sialkot, Sialkot 51300, Pakistan; 3 School of Pharmacy, Nanjing University of Chinese Medicine, Nanjing 210023, China; 4 Department of Pharmaceutical Chemistry, College of Pharmacy, King Saud University, P.O. Box 2457, Riyadh 11451, Saudi Arabia; 5 Peptide Chemistry Department, Chemical Industries Research Institute, National Research Centre, Cairo 12622, Egypt; 6 Department of Chemistry, Faculty of Science, Port Said University, Port Said 42526, Egypt

**Keywords:** Mannich bases, isoflavenes, anticancer, DFT, docking

## Abstract

Isoflavenes have received the greatest research attention among the many groups of phytoestrogens. In this study, various isoflavene-based Mannich bases were selected for their theoretical studies. The purpose of this research was to discover the binding potential of all the designated Mannich bases acting as inhibitors against cancerous proteins EGFR, cMet, hTrkA, and HER2 (PDB codes: 5GTY, 3RHK, 6PL2, and 7JXH, respectively). For their virtual screening, DFT calculations and molecular docking studies were undertaken using in silico software. Docking studies predicted that ligands 5 and 15 exhibited the highest docking score by forming hydrogen bonds within the active pocket of protein 6PL2, ligands 1 and 15 both with protein 3RHK, and 7JXH, 12, and 17 with protein 5GTY. Rendering to the trends in polarizability and dipole moment, the energy gap values (0.2175 eV, 0.2106 eV) for the firm conformers of Mannich bases (1 and 4) replicate the increase in bioactivity and chemical reactivity. The energy gap values (0.2214 eV and 0.2172 eV) of benzoxazine-substituted isoflavene-based Mannich bases (9 and 10) reflect the increase in chemical potential due to the most stable conformational arrangements. The energy gap values (0.2188 eV and 0.2181 eV) of isoflavenes with tertiary amine-based Mannich bases (14 and 17) reflect the increase in chemical reactivity and bioactivity due to the most stable conformational arrangements. ADME was also employed to explore the pharmacokinetic properties of targeted moieties. This study revealed that these ligands have a strong potential to be used as drugs for cancer treatment.

## 1. Introduction

Globally, cancer is the second-leading cause of death after cardiovascular diseases [1,2,3]. Carcinogenesis is a multistage process in which changes in tissue frames and cell phenotypes can instigate local regions of hypoxia. This stimulates the survival and growth of tissue stem cells and the formation of precancerous and cancerous lesions. Cancer is a complex and aggressive disease that is strenuous to treat due to its non-static progress, mutations in hundreds of genes, and widespread effects [4]. There are various conventional methods to treat cancer, including radiotherapy and chemotherapy, and many advanced approaches such as nanoparticles, sonodynamic therapy, ablation therapy, targeted therapy, natural antioxidants, chemodynamic therapy, and stem cell therapy [5]. Despite the striking developments in the methods of cancer healing in recent decades, chemotherapy persists as the focal method for cancer treatment. Expeditiously increasing numbers of biomedical studies are focused on designing chemotherapeutics that are proficient at evading or disabling MDR [6]. To treat cancer, anticancer drugs that act as multipurpose swords are better than drugs that only act on a single aspect of the disease [7].

In most Western nations, hormone-dependent malignancies, including breast and prostate cancer, are on the rise. By the age of 85, one in eight Australian women will have been diagnosed with breast cancer. A woman’s chance of developing breast cancer rises with age, and the majority of those diagnosed are between the ages of 50 and 69. Over 297,790 women had breast cancer diagnoses in 2023, and 43,700 people passed away from the disease [8]. Invasive breast cancer will be identified in 2800 males in the United States in 2023, according to estimates. Prostate cancer is more likely to occur as people age, with men over 65 accounting for 85% of cases [9]. Plants, including beans, grains, vegetables, fruit, and seeds, contain phytoestrogens, which are estrogen-like compounds. Low occurrences of hormone-dependent malignancies, including prostate and breast cancer, have been associated with Asian people’s high dietary intake of phytoestrogens, which is partly attributable to their high soy food consumption. Due to their pronounced estrogenic effects, isoflavenes have received the greatest research attention among many groups of phytoestrogens. In clinical studies, the synthetic isoflavene derivative phenoxodiol has been evaluated for the treatment of drug-resistant prostate (NCT00557037) and ovarian (NCT00382811) cancers. Its capacity to cause mitotic arrest, terminal differentiation, and apoptosis in cancer cells has been linked to its broad range of anticancer efficacy [10,11,12].

The biologically active Mannich bases are a structurally diverse family of chemical compounds formed by the addition of an aminomethyl functional group via the Mannich reaction, also known as amino alkylation reactions, involving active hydrogen, an amine, and an aldehyde. It has a wide range of uses in the treatment of natural macromolecules such as leather, paper, textiles, cosmetics products, analytical reagents, paint, water treatment, as an additive in the petroleum industry, and the preparation of synthetic polymers [13,14,15]. However, pharmaceutical chemistry is a significant field for Mannich bases, as evidenced by the multiple articles published on the importance and uses of this active class each year [16,17]. In the first place, Mannich bases may possess interesting biological properties, many of which have yet to be discovered through diligent screening. Secondly, aminomethylation of drugs also improves their bioavailability. The Mannich bases potentially help increase medication distribution in humans [18,19,20]. The drug’s hydrophilic characteristics might be improved by introducing a polar molecule into the parent structure through the Mannich reaction. Furthermore, by using a suitable amine reagent in the Mannich process, the drug’s lipophilic characteristics can be boosted. Moreover, aminomethylated pharmaceuticals can be used as prodrugs via deamination or deaminomethylation under controlled hydrolytic conditions to release the active component [21,22].

Mannich bases have been shown in several studies to have major biological effects such as anticancer, antibacterial, analgesic, antiviral, anti-inflammatory, anticonvulsant, anti-Alzheimer, anti-HIV, antimalarial, antioxidant, anthelmintic, etc. A variety of derivatives of ketonic Mannich bases were evaluated for their cytotoxicity against Jurkat cells and androgen-independent prostate cancer cells (PC-3) [23]. Analogs of Mannich bases of heterocyclic chalcone have been studied for their cytotoxic activity against four human cancer cell lines, namely, PC-3, MCF-7, nasopharyngeal carcinoma (KB), and resistant nasopharyngeal carcinoma (KB-VIN) [24]. The Mannich bases of lawsone, another phenolic quinone that occurs naturally, as well as their Pt(II) complexes, are highly cytotoxic toward six cancer cell lines: MDA-MB-435 (melanoma), HL-60 (promyelocytic leukemia), HCT-8 (colon), SF-295 (brain), OVCAR-8 (ovary), and PC-3 (prostate). They worked through the inhibition of ethidium bromide intercalation into DNA [25]. There has been some evidence suggesting that C-type Mannich bases of indole derivatives act as potent inhibitors of isoprenylcysteine carboxyl methyltransferase (Imct), an enzyme that provokes modification in the post-translational proteins that are engaged in the regulation of cell growth; thus, it could be therapeutically targeted in oncogenesis [26].

**Figure 1:**
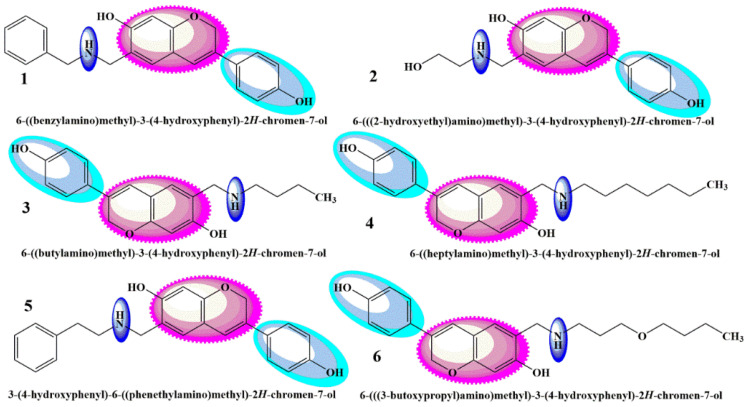
Class 1 aminomethyl-substituted isoflavenes [27].

**Figure 2:**
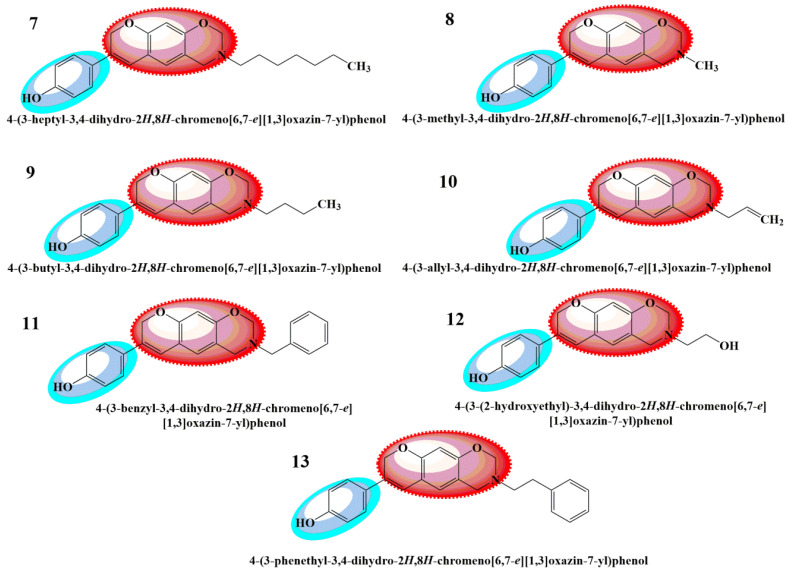
Class 2 benzoxazine-substituted isoflavenes [27].

**Figure 3:**
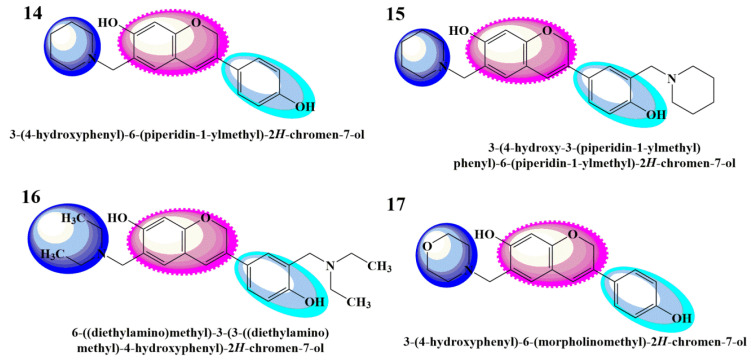
Class 3 isoflavenes with a tertiary amine [27].

As there is still a need to uncover the anticancer agents that possess high efficacy and the fewest side effects, the present research work was carried out to theoretically study the biologically active aminomethyl-substituted (Figure 1) and benzoxazine-substituted isoflavene (Figure 2), and isoflavenes with tertiary amine-based Mannich bases (Figure 3) (reported by Yilin et al. as potential anticancer agents), as shown in Figure 1, Figure 2 and Figure 3, respectively [27]. Their in silico anticancer properties were also evaluated through docking studies of the ligand binding domain of the androgen receptor prostate cancer mutant H874Y (PDB ID: 2Q7L) connected to testosterone and a TIF2 Box3 coactivator peptide 740–753. For experimental analysis, the selected Mannich bases were analyzed through DFT and molecular docking. The Hartree–Fock method, simulation techniques, molecular mechanics, post-Hartree–Fock studies using MP2 and MP3, and density functional theory (DFT) were all used to report various structural and electronic characteristics of selected organic molecules [28]. Using DFT B3LYP/6-31G(p,d) basis sets, the structural characterizations of the selected compounds were evaluated. Other theoretical predictions, such as chemical reactivity, natural bond orbital analysis, equilibrium geometry, frontier molecular orbital analysis, and molecular electrostatic potential, were investigated and computed using DFT methods. The exploration and explanation of pharmacokinetic processes through the characterization of absorption, distribution, metabolism, and excretion (ADME) features were studied to enable the provision of safety considerations for a novel medicine on the basis of which risk-based evaluations may be made.

## 2. Results

### 2.1. DFT Studies

**Figure 4:**
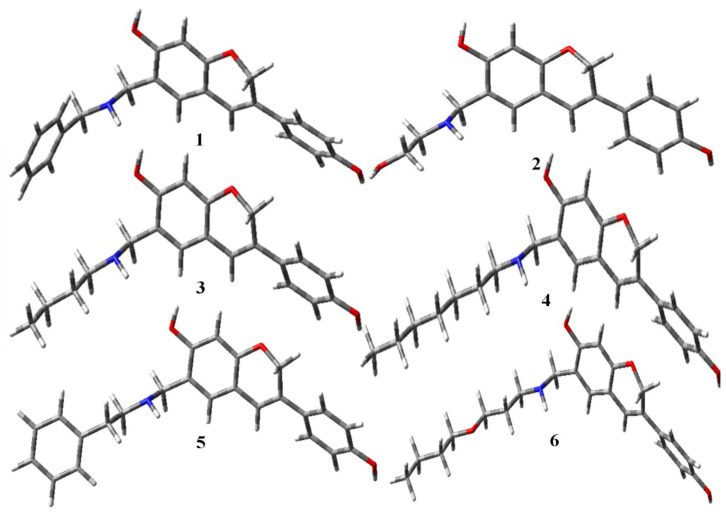
Optimized structures of aminomethyl-substituted isoflavenes.

**Figure 5:**
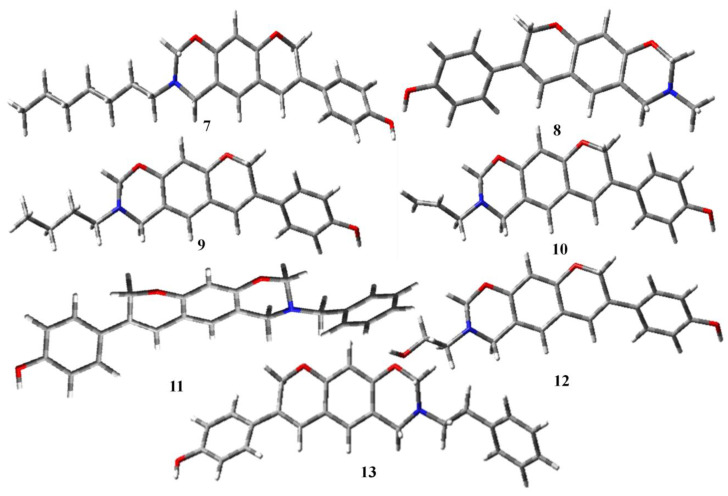
Optimized structures of benzoxazine-substituted isoflavenes.

**Figure 6:**
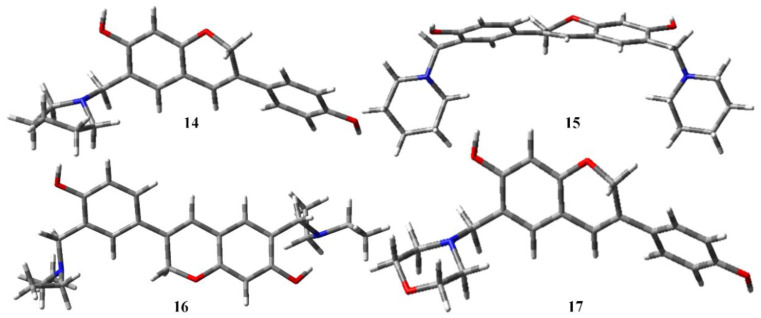
Optimized structures of isoflavenes with a tertiary amine.

Moving the atoms in a molecule to obtain the most stable configuration with the least amount of ground state energy is known as geometry optimization. Using density functional theory (DFT) in the gas phase, the electrical characteristics were theoretically determined using the DFT/B3LYP/6-31G level (Table 1). The optimized geometries of all the selected compounds are given in Figure 4, Figure 5 and Figure 6 [29].

#### 2.1.1. Global Chemical Reactivity Descriptors

The chemical reactivity descriptors, including ionization potential, chemical hardness and softness, total energy, and electrophilicity, were calculated using DFT.

HOMO is the highest occupied molecular orbital, and LUMO is the lowest unoccupied molecular orbital. The HOMO and LUMO energies are related to the ionization energy and electron affinity of the molecule [30].

Koopmans’ theorem equation: A = −E_HOMO_ and I = −E_LUMO_.

The energy difference gives information about the reactivity of the molecules. The larger gap indicates that the molecule is highly stable and less reactive, while the lower energy gap predicts that the molecule is least stable and highly reactive [30]. Compound **9** (0.2214 eV) exhibits the highest energy gap, indicating that it is the most stable and least reactive, while compound **16** (0.1431 eV) shows the least energy gap and acts as the most reactive and least stable species. The lowest ionization value of compound **16** (0.1725 eV) indicates that it is more reactive and easier to ionize. The negative electronegativity of a structure is referred to as the electronic chemical potential and denoted as µ.

µ = (E_HOMO_ − E_LUMO_)/2

It measures the escaping capacity of an electron from a system. A higher value indicates that a molecule is more reactive and unstable. The electrophilicity index is a measurement of the capacity of a system to attract an electron [31]. It is calculated using the following formula:

ω = µ^2^/2η

For all the investigated compounds, the electrophilicity index ranged from 0.0712 to 0.0984 eV, so they are nucleophilic due to the low value of the electrophilicity index. Chemical hardness is related to the stability and reactivity of a chemical system. It estimates the extent of resistance to changes in the distribution of electrons [32].

η = (I − A)/2

Compound **9** is the chemically hardest (0.1107 eV) among all the compounds, while compound **7** is the least hard, with a chemical hardness value of 0.0716 eV.

#### 2.1.2. Frontier Molecular Orbitals

The ability of a molecule or system to donate and take electrons is represented by its HOMO and LUMO molecular orbitals. One of the most crucial factors that influence how a molecule behaves, how hard or soft it is, and how reactive it is is the difference in energy between these molecular orbitals. Additionally, this energy gap’s value is a characteristic that has an impact on optical polarizability. Soft molecules can be polarized more readily than hard molecules because they require less energy to be excited. The anticancer and antimutagenic actions of the selected compounds under research, which had not previously been reported in the literature, were, respectively, explained by the HOMO–LUMO energy gaps and the percent atom contributions to the HOMO. For a very long period of time, the comparative reactivity and bioactivity of organic compounds were assessed using frontier molecular orbitals (FMOs). For the FMOs, the lowest unoccupied molecular orbital (LUMO) and the highest occupied molecular orbital (HOMO) are both responsible. The HOMO–LUMO energy gap aids in characterizing the chemical reactivity, bioactivity, and kinetic stability of the molecule, whereas the HOMO and LUMO energies describe the capacity to provide and take electrons, respectively. A molecule with a short HOMO–LUMO energy gap is more polarizable and often has higher chemical and biological activity as well as lower kinetic stability [33].

**Figure 7:**
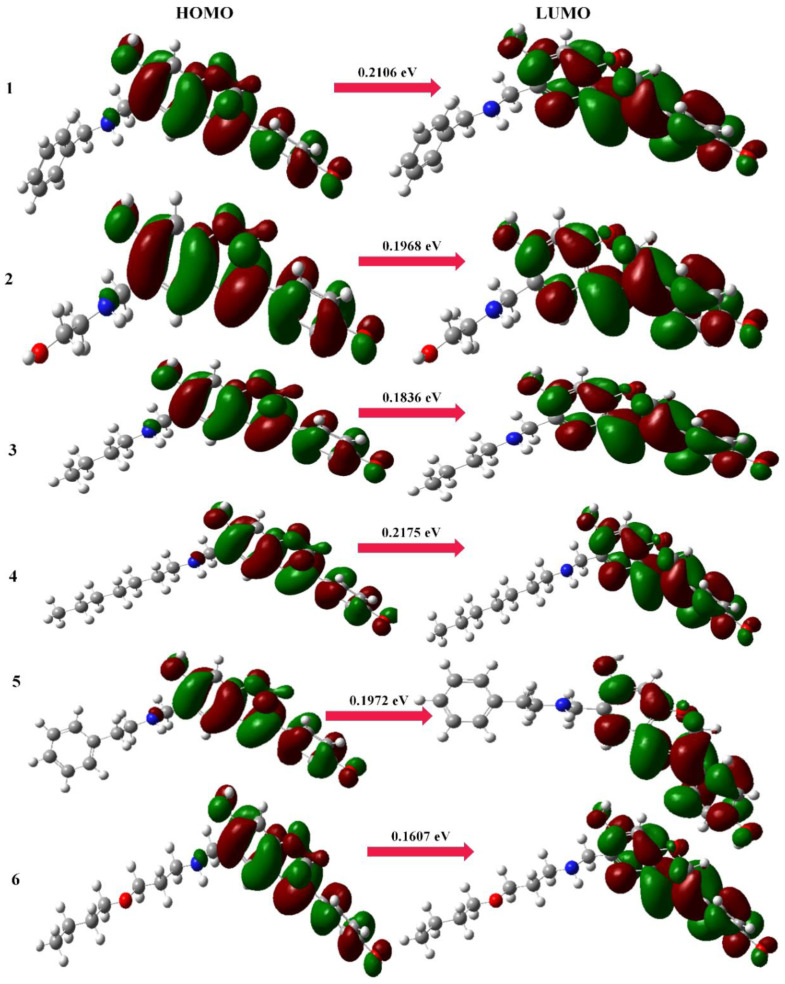
HOMO and LUMO maps of aminomethyl-substituted isoflavenes.

**Figure 8:**
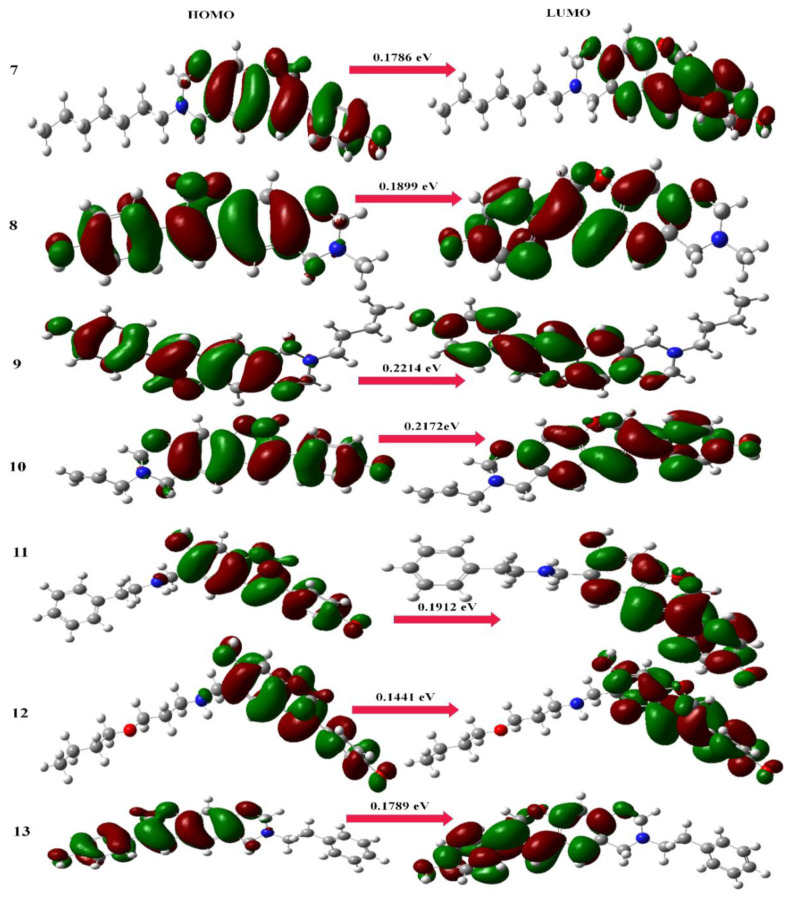
HOMO and LUMO maps of benzoxazine-substituted isoflavenes.

**Figure 9:**
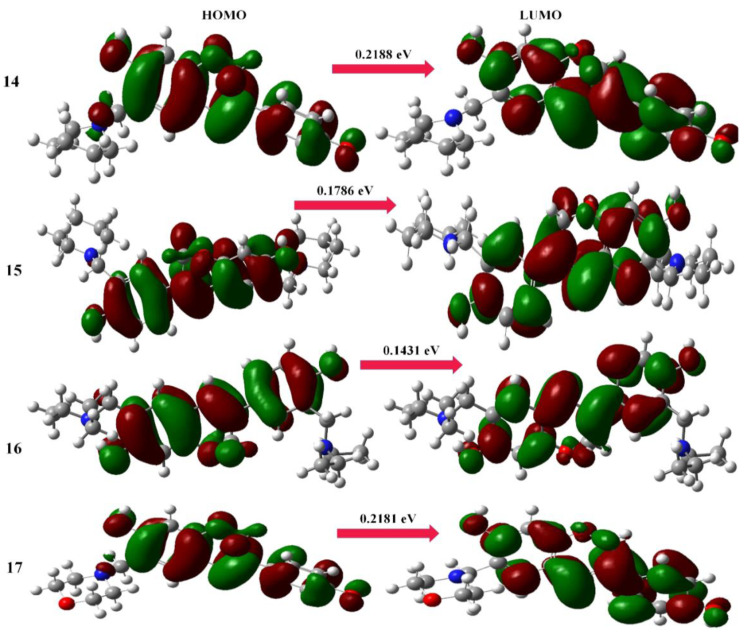
HOMO and LUMO maps of isoflavenes with a tertiary amine.

At the B3LYP/6-311G (d,p) level, the HOMO–LUMO energy gaps for the conformational spaces of selected Mannich bases (aminomethyl-substituted isoflavenes) were computed (Figure 7). According to the trends in dipole moment and polarizability, the energy gap values (0.2175 eV and 0.2106 eV) for the stable conformers of Mannich bases (**1** and **4**) reflect the increase in chemical reactivity and bioactivity [28]. The energy gap values (0.2214 eV and 0.2172 eV) of benzoxazine-substituted isoflavene-based Mannich bases (**9** and **10**) reflect the increase in chemical reactivity and bioactivity due to the most stable conformational arrangements (Figure 8). The energy gap values (0.2188 eV and 0.2181 eV) of isoflavenes with tertiary amine-based Mannich bases (**14** and **17**) reflect the increase in chemical reactivity and bioactivity due to the most stable conformational arrangements (Figure 9).

#### 2.1.3. Molecular Electrostatic Potential

The electrophilic and nucleophilic areas of a molecule may be identified using a molecular electrostatic surface map and a color-coded system (red, orange, yellow, green, and blue). Red represents regions that are electron-rich in this color scheme, whereas blue depicts parts that are electron-poor. In comparison to red and neutral zones, yellow and green signify less favorable locations. The following is the correspondence between the surface’s electrostatic potential and the color scheme: red denotes areas with negative electrostatic potential and electron abundance; white denotes areas with neutral electrostatic potential and zero electron abundance; and blue denotes areas with positive electrostatic potential and electron abundance. Regions of negative potential are generally associated with the lone pair present on electronegative atoms [34]. While the blue color indicates the positively charged region is attracted to electrophiles, the green-colored area indicates zero potential. The color grading of MEP shows the molecular shape and size, as well as neutral, negative, and positive potential [35]. This can be seen from the MEP map of the positive potential spread on the hydrogen atoms and the negative potential on the electronegative atoms. It is noteworthy that the gas phase MEP surfaces fall between −6.649 × 10^−2^ eV and +6.649 × 10^−2^ eV.

**Figure 10:**
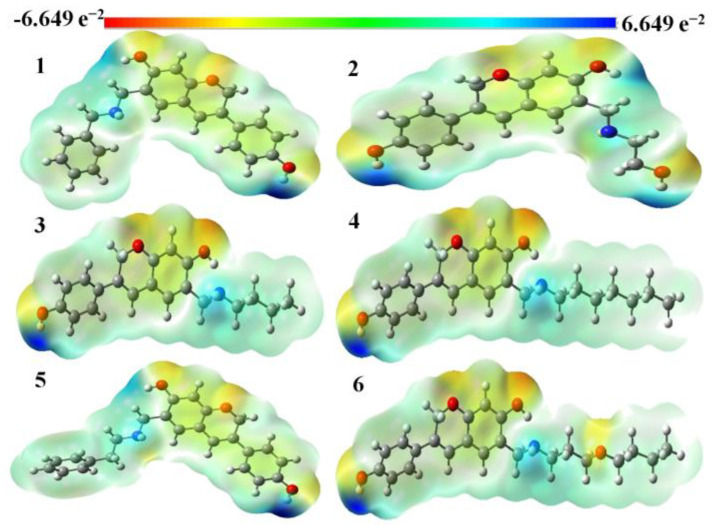
MEP diagrams of aminomethyl-substituted isoflavenes.

The molecular electrostatic potential map of aminomethyl-substituted isoflavenes during the gas phase is shown in Figure 10 [15]. The most electrophilic area is concentrated on the oxygen atoms of the hydroxyl groups and the chromen ring of the selected Mannich Bases, which are located in the red region. The yellow (slightly electron-rich) portion of the title molecules contains the C atoms of the chromen and phenyl rings. The blue area with few electrons also contains N and H atoms. We may infer that the title molecules can enter a reaction, particularly through N atoms, based on the data from the MEP map [36].

**Figure 11:**
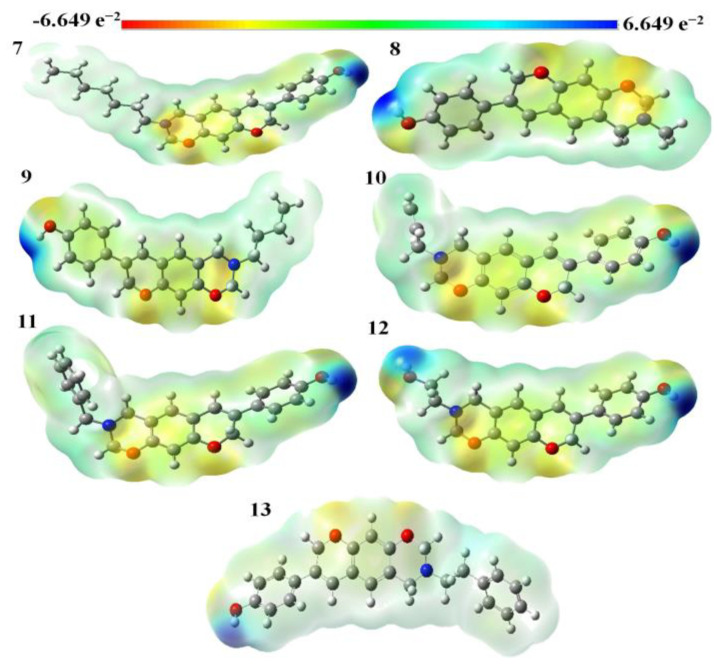
MEP diagrams of benzoxazine-substituted isoflavenes.

The molecular electrostatic potential map of benzoxazine-substituted isoflavenes during the gas phase is shown in Figure 11. The most electrophilic area is concentrated on the oxygen atoms of the chromeno-oxazine ring of the selected Mannich bases, which are located in the red region. The yellow (slightly electron-rich) portion of the title molecules contains the C atoms of the chromeno-oxazine and phenyl rings and the oxygen atom of the hydroxyl group. The blue area with few electrons also contains N and H atoms. Based on the information from the MEP map, we may deduce that the title molecules can enter a reaction, notably through N atoms.

**Figure 12:**
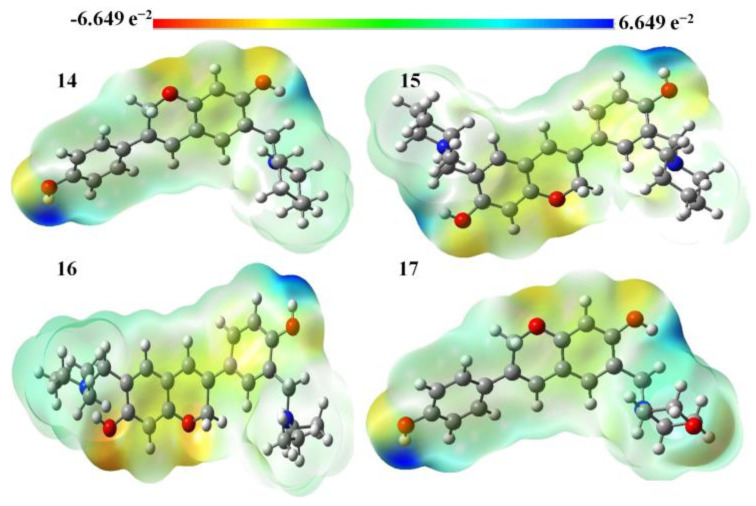
MEP diagrams of isoflavenes with a tertiary amine.

Figure 12 depicts the molecular electrostatic potential map of the tertiary amine-containing isoflavenes during the gas phase. The oxygen atoms of the hydroxyl group and chromen ring of the chosen Mannich bases are concentrated in the red zone, which is the most electrophilic region. The chromen and phenyl rings’ C atoms, as well as the oxygen atom from the hydroxyl group, are located in the yellow (somewhat electron-rich) region of the title molecules. N and H atoms are also present in the blue region with few electrons. We may infer that the title molecules can enter a reaction, particularly through N atoms, based on the data from the MEP map.

### 2.2. Docking Studies

In drug development, molecular docking is crucial because it aids in the prediction of inhibition. It is the primary factor in in silico investigations that leads to the discovery of novel pharmaceutical substances. However, docking makes it easier to comprehend the mode of action between any ligand and protein while also demonstrating the ligand’s interactions with the protein. The default docking setup settings were applied to the ligand docking experiment in Maestro 11.2. A gliding experiment makes predictions about the binding energies of ligands in the protein’s active region. The two top-scoring docking compounds’ 3D and 2D visuals were obtained using Maestro. With a certain protein, each ligand provided a distinct value in tabular form; the effectiveness of docking is seen in the project table of Maestro 11.2.

Using docking studies on the receptors cMet, EGFR, HER2, and hTrkA (PDB codes: 3RHK, 5GTY, 7JXH, and 6PL2, respectively), the reference drugs (Erlotinib, Neratinib, and Tepotinib) and chosen Mannich base compounds **1**–**17** were evaluated for their capacity to bind to the chosen receptors. The importance of the four chosen receptors in the initiation and progression of cancer is well understood.

**Figure 13:**
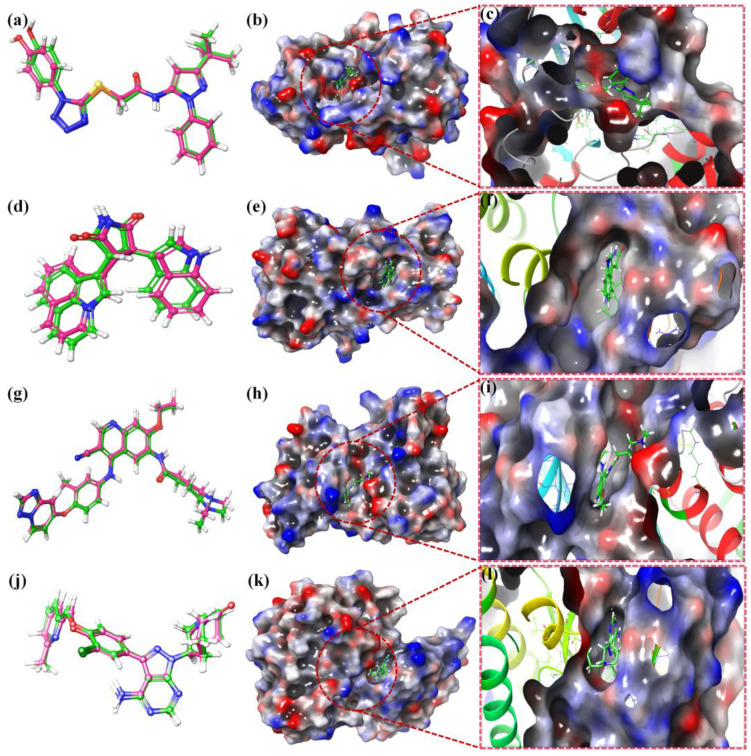
Native co-crystallized (green) and redocked (red) inhibitors at the 6PL2, 3RHK, 7JXH, and 5GTY receptors, superimposed in a 2D and 3D overlay diagram for program confirmation (**a**,**b**,**d**,**e**,**g**,**h**,**j,k**). Surface rendering of ligands with the examined compounds overlaid at the binding location for the catalytic domain (**c**,**f**,**i**,**l**).

First, two validation procedures were used to demonstrate the Schrodinger program’s reliability in producing accurate docking findings. This was proven by redocking the respective 6PL2, 3RHK, 7JXH, and 5GTY receptors with each of the two co-crystallized inhibitors (OOM, M97, VOY, and 816). The successful execution of the docking process was ensured by the low acquired values of RMSD (1.66 and 1.63, respectively), which reflect the root mean square deviation between the native and redocked positions of the co-crystallized inhibitor. Additionally, Figure 13 showed a 3D superimposition of the native co-crystallized and redocked inhibitors (816, OOM, M97, and VOY).

OOM was discovered to create two H-bonds with GLU590 and ASP668, and one pi-H bond with PHE669 amino acids at 1.97, 2.12, and 3.41, respectively, as a native co-crystallized inhibitor of the 6PL2 receptor. However, at 2.16, 2.12, 2.89, and 2.96, respectively, M97, the co-crystallized inhibitor of the 3RHK receptor, established two H-bonds with MET1160 and PRO1158, and one H-bond with PHE1223. In addition, at 2.13 and 2.18, respectively, MET801 and ASP863 amino acids formed two H-bonds with VOY, the co-crystallized inhibitor of the 7JXH receptor. The co-crystallized 5GTY receptor inhibitor 816 formed three H-bonds with the amino acids GYS797, MET793, and GLN791 at 2.98, 1.94, and 2.22, respectively. The architecture of the catalytic site and surrounding areas was both electrically and sterically consistent with all ligands (Figure 13c,e,h,k).

**Figure 14:**
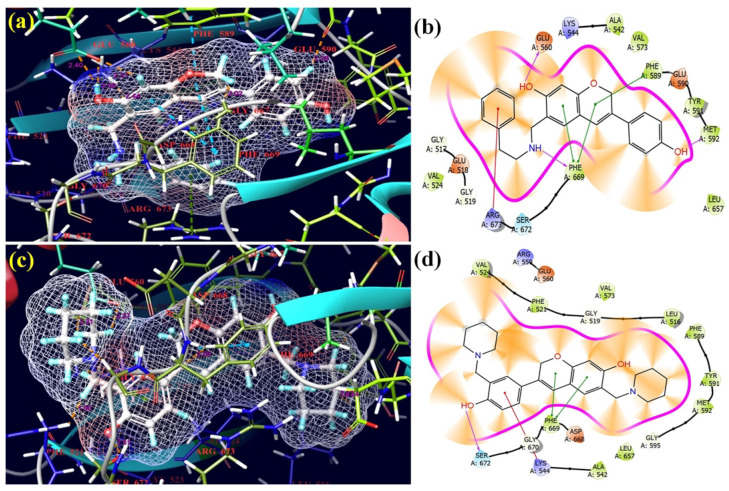
3D and 2D pictorials of molecular docking for cancer protein and compound **5** (**a**,**b**), and compound **15** (**c**,**d**) with protein 6PL2.

**Figure 15:**
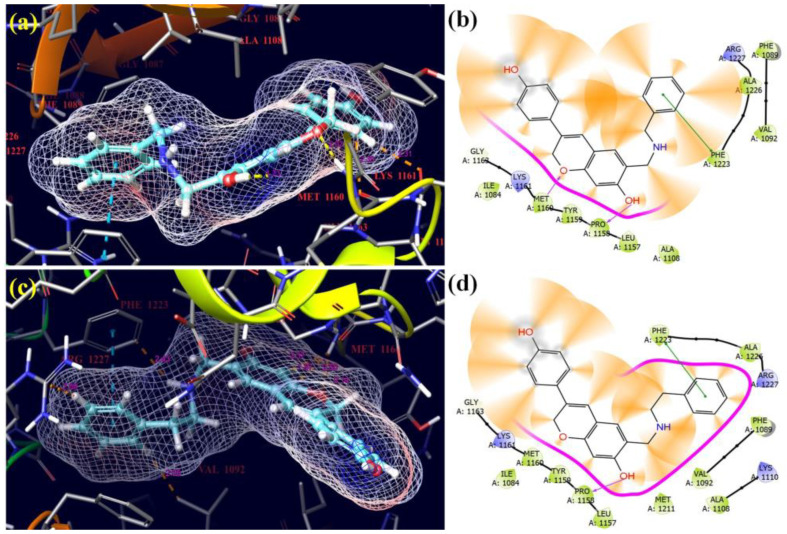
3D and 2D pictorial of molecular docking for cancer protein and compound **1** (**a**,**b**), and compound **5** (**c**,**d**) with protein 3RHK.

**Figure 16:**
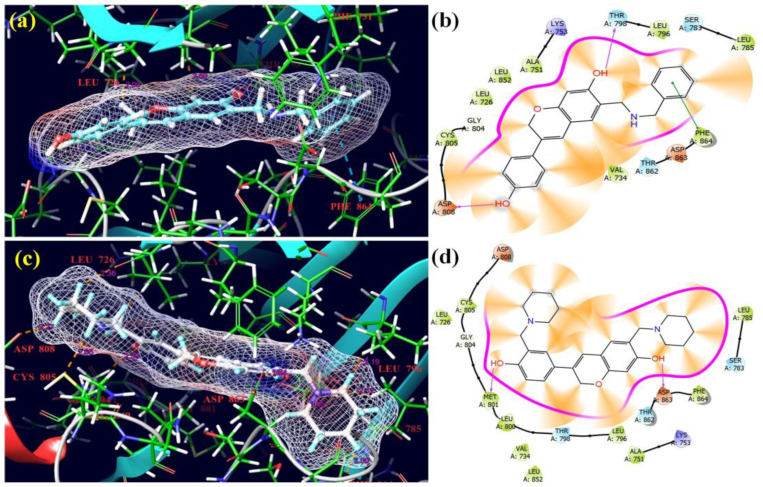
3D and 2D pictorials of molecular docking for cancer proteins and compound **1** (**a**,**b**), and compound **15** (**c**,**d**) with protein 7JXH.

**Figure 17:**
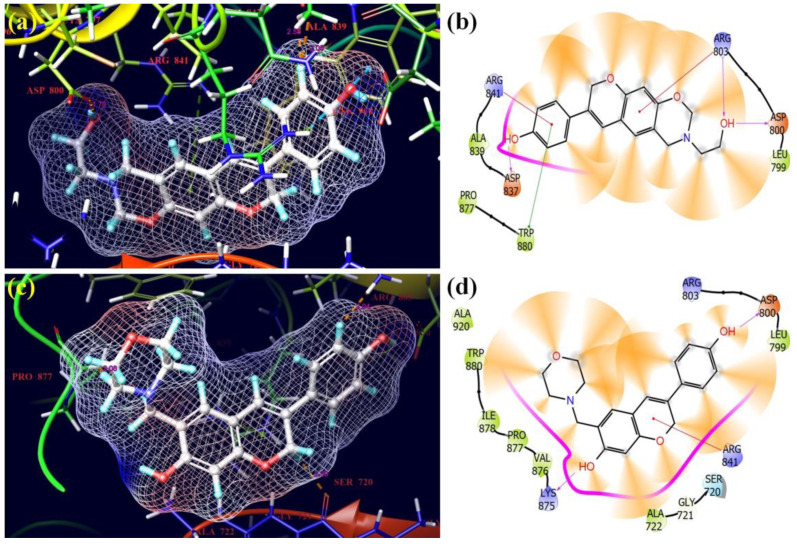
3D and 2D pictorials of molecular docking for cancer proteins and compound **12** (**a**,**b**) and compound **17** (**c**,**d**) with protein 5GTY.

After the docking methodology had been verified, compounds **1** to **17** were docked, and their docking scores were evaluated. The binding similarities of the selected compounds, specifically Erlotinib, Tepotinib, and Neratinib (Table 2), whose in vivo studies are reported against anticancer agents, were then compared to those of known inhibitors of the 6PL2 (Figure 14), 3RHK (Figure 15), 7JXH (Figure 16), and 5GTY (Figure 17) receptors. Compound **13** is an extremely effective inhibitor, according to the stated data from in vitro investigations [27]; our in silico results also matched the reported data. Compound **1** is effective against the 3RHK protein that was chosen, whereas compound **17** is effective against 5GTY, compound **7** against 7JXH, and compound **5** against 6PL2. Table 2 lists the docking scores, and snapshots of the docked compounds, which revealed the lowest docking scores, were also provided. According to the projected binding interactions for the 3RHK receptor, the docking scores and glide energies (kcal/mol) were between 5.953 and 9.893, and –39.325 and –46.180, respectively, which indicated good to exceptional interactions compared to interactions with typical medicines.

The docking scores and glide energies (kcal/mol) for the anticipated binding interactions for the 5GTY receptor were between −2.968 and −4.127, and −25.386 and −26.422, respectively, which pointed to lower-level interactions compared to those of typical drugs. The docking scores and glide energies (kcal/mol) for the expected binding interactions of all selected Mannich bases for the 6PL2 receptor were between −6.088 and −10.655, and −43.757 and −51.955, respectively, which are far better than the control drugs (Erlotinib, Tepotinib, and Neratinib). These values pointed to extraordinary interactions compared to those of standard drugs. The docking scores and glide energies (kcal/mol) ranged from −4.942 to −8.567, and −32.092 to −53.848, respectively, which showed that the anticipated binding interactions for the 7JXH receptor represented exceptional interactions compared to interactions with conventional drugs [37].

The ligands **5** and **15** had the lowest docking scores and glide energies; these derivatives were successfully placed into the receptor’s binding pockets of 6PL2 (Figure 14). While the phenyl and chromen rings of isoflavene formed π–π interactions with PHE-589 and PHE-669 residues with a distance of 4.24 Å and 5.09 Å, respectively, and the nitrogen of an amino group created hydrogen bonds with a distance of 2.09 Å with the receptor’s (PHE-669) residue in ligands **5**, ligand **15**′s structure was more flexible and demonstrated a better affinity with the receptor (PHE-669) and the benzene ring with a distance of 4.61 Å and 4.91 Å, respectively, and the hydroxyl group showed an interaction with LYS 544 and hydrogen bonding with SER 672 residues with 5.24 Å and 1.57 Å, respectively. All selected isoflavene-based Mannich bases have substantially higher binding affinities than Tepotinib, Erlotinib, and Neratinib. These selected Mannich bases are the most favorable candidates against the 6PL2 cancer protein.

With the lowest docking scores and glide energies, ligand derivatives **1** and **5** successfully fitted into the receptor’s binding pockets (3RHK) (Figure 15). The receptor residues PHE-1223 with 4.00 Å were joined by π–π interactions with benzene rings in ligand **1**. The hydroxyl and oxygen of benzene and the chromen ring of isoflavene of compound **1** created hydrogen bonds with the receptor residues PRO-1158 with 1.73 Å and MET-1160 with 2.02 Å. In compound **5**, the benzene rings made π–π interactions with receptor residues PHE-1223 with 4.01 Å, and the hydroxyl of the benzene ring of isoflavene created hydrogen bonds with the receptor residues PRO-1158 with 1.84 Å. All selected Mannich bases showed excellent activity against the 3RHK cancer protein as compared to three control drugs: Tepotinib, Erlotinib, and Neratinib.

With the lowest docking scores and glide energies, ligand derivatives **1** and **15** successfully fitted into the receptor’s binding pockets (7JXH) (Figure 16). The receptor residues PHE-864 with 5.04 Å were joined by π–π interactions with benzene rings in ligand **1**. The hydroxyl groups of the benzene ring of isoflavene and the benzene ring of the side chain of compound **1** created hydrogen bonds with the receptor residues THR-796 with 2.17 Å and ASP-808 with 1.77 Å. In compound **15**, hydroxyl groups of the benzene ring of isoflavene and the benzene ring of the side chain created hydrogen bonds with the receptor residues ASP-863 with 1.64 Å and MET-801 with 2.07 Å, respectively. All selected Mannich bases showed wonderful activity against the 7JXH cancer protein as compared to three control drugs: Tepotinib, Erlotinib, and Neratinib [38].

The most fascinating compounds when examining their binding attractiveness to the chosen receptors were **12** and **17** (Figure 17). Despite having the lowest docking scores and glide energies, ligands **12** and **17** were successfully inserted into the receptor’s binding pockets (5GTY). ARG-803 and ARG-841 with 5.54 Å and 3.57 Å, respectively, and TRP-880 residues of the receptor with 4.09 Å formed π–π interactions with the phenyl ring of isoflavene and the benzene ring of the side chain of **12**, respectively. The OH groups of **12** also underwent hydrogen bonding with ARG-803 with 1.88 Å, ASP-800 with 1.70 Å, and ASP-837 with 2.01 Å residues of the 5GTY protein. In compound **17**, the hydroxyl groups bonded through hydrogen bonding with ASP-800 with 1.98 Å and LYS-875 with 2.23 Å, and the chromen ring of isoflavene showed π–π interactions with ARG-841 with 4.92 Å. Overall, however, the binding affinities of the selected Mannich bases were inferior to those of the standard drugs.

5GTY: 3>, 6>, >5>, >2, >4, >1

3RHK: 1>, 5>, 4>, 6>, 3>, 2

7JXH: 1, >5, >6, >3, >2, >1

6PL2: 5, >2, >6, >4, >3, >1

Class 1 of the selected compounds having aminoalkyl substituents showed good-to-excellent potential/docking scores against the selected proteins. Compound **5** also showed the highest potential against 6PL2 because it has a phenyl moiety similar to compound **1**, which also has good potential. We suggested here that if we introduced substituents having a hydroxyl group on the aromatic moiety of compound **5**, this would increase its potential because isoflavenes are structural isomers of flavonoids. The phenolic group of flavonoids is mainly responsible for their anticancer potential. Compound **6** also has good scores against all selected proteins. C–H…O hydrogen bonds form inversion dimers, which are linked to each other by additional hydrogen bonds and involved in the formation of three-dimensional networks. In vitro, the anti-proliferative properties of class 2 Mannich bases in the case of compound **5** are also in agreement with docking studies.

5GTY: 12 > 7 > 11 > 13 > 8 > 10 > 9

3RHK: 9 > 11 > 10 > 12 > 7 > 8 > 13

7JXH: 7 > 9 > 12 > 10 > 11 > 13 > 8

6PL2: 9 > 10 > 12 > 8> 13 > 11 > 8

Selected class 2 compounds also showed good potential/docking scores against all selected proteins. Compound **9** showed an excellent potential/docking score against 3RHK and 6PL2 proteins due to the substitution of the alkyl group; a similar trend was also shown by compound **7** against 7JXH protein due to alkyl substitution. Compound **12** also showed good potential for 5GTY due to the –OH group at the end of the alkyl chain, which caused hydrogen bonding. Thus, here we suggest that the anticancer potential of the class 2 compounds can be increased by introducing alkyl chains ending with –OH or –NH2, which may provide options for hydrogen bonding and also cause insertion into the protein binding pockets. In vitro, the anti-proliferative properties of class 2 Mannich bases in the case of compound **7** are also in agreement with docking studies.

5GTY: 15 > 14 > 16 > 17

3RHK: 17 > 15 > 16 > 14

7JXH: 14 > 15 > 16 > 17

6PL2: 15 > 16 > 17 > 14

Selected class 3 compounds also showed excellent potential/docking scores against all selected proteins. In this class, compound **15** is the most prominent for all proteins, which may be due to the piperidin-1-ylmethyl moiety, which is also present in compound **14** and is active for the 7JXH protein. In vitro, the anti-proliferative properties of class 2 Mannich bases in the case of compound **15** are also in agreement with docking studies. Thus, here we suggest that the piperidin-1-ylmethyl moiety can be further modified to enhance the anticancer potentials of class 3 compounds.

### 2.3. ADME Results

As part of the ADME study, the Maestro software was used to calculate physicochemical parameters, including molecular weight, dipole moment, volume, polar surface area, solvent accessible surface, brain/blood partition coefficient, human oral absorption, human oral absorption percentage, MDCK cell permeability, rule of five, and rule of three of the twelve molecules. The results of these computations are listed in Table 3. The rule of three and the rule of five are the two most important parameters out of all the calculated parameters. These parameters must have a numerical value between 0 and 3 for the rule of three and between 0 and 4 for the rule of five. Using Lipinski’s rule of five, which considers a compound’s molecular weight (MW), lipophilicity (log P), number of hydrogen bond acceptors (HBA), and number of hydrogen bond donors (HBD), all compounds were assessed for their drug-likeness. There were no violations of Lipinski’s rule in ten compounds. Additionally, it has been suggested that compounds with fewer violations of Jorgensen’s rule of three (QPlogS > 5.7, QPPCaco > 22 nm/s, and # Primary Metabolites 7) are more likely to be consumed. It was discovered that all compounds adhered to Jorgensen’s rule of three. Absorption is influenced by the drug’s solubility, permeability, interactions with transporters, and metabolic enzymes in the gut wall, among other factors. Most compounds showed high projected qualitative human oral absorption values and 100% predicted qualitative human oral absorption.

Notably, drug binding to plasma greatly limits the amount of the drug in blood circulation; hence, the less bound a drug is, the better its capacity to disperse or cross cell membranes. In the requisite QPlogKhsa range of 1.5 to 1.5 (prediction of binding to human serum albumin), almost all compounds are found. This suggests that most chemicals are likely free to circulate inside the circulation and hence reach the target area. The findings of ADME showed that almost all medicines had properties seen in other pharmaceuticals [39,40].

## 3. Materials and Methods

### 3.1. DFT Calculations

Isoflavene-based Mannich bases were selected from the literature to be theoretically investigated. ChemDraw 12 Pro was used to draw the structures of the molecules. Then, these structures were saved as mol files and opened in GaussView. Calculations were run by selecting optimization using the DFT and B2LYP sets [41]. After completion of the Gaussian job, two output files were generated, a chk file and a gif file. Next, FMO and MEP structures were drawn using a chk file [42]. Modeling of the targeted compounds was executed by GaussView 16 software, and Gaussian 09 software was used for calculations. Molecular electrostatic potential (MEP) and HOMO and LUMO orbitals were generated from optimized structures.

### 3.2. Molecular Docking

The structures of selected Mannich bases were drawn using ChemDraw 12 Pro and used as a mol2 file. These structures were then incorporated into Maestro. From the ligand prep menu, all the ligands were selected and prepared, and their confirmations were optimized [43]. The androgen receptor prostate cancer mutant H874Y (PDB ID: 2Q7L) connected to the testosterone protein structure was downloaded from the RCSB Protein Data Bank. For the preparation of proteins, the “Protein Preparation” wizard was used. The bond order was assigned, and a hydrogen atom was added by defining the co-crystallization at the active site of the protein [38]. In the Molecular Docking step, the grid-generated protein and prepared ligands were added to the Ligand Preparation menu. Then, the most favorable binding mode of ligands with the selected protein was checked out, and their docking scores were added to table. ADME was performed by selecting “ADME Quik prop” in Maestro, Schrödinger, LLC, New York, NY, 2023 [44].

## 4. Conclusions

Mannich bases are a very important class due to their versatile use in drug formation. The DFT/B3LYP/6–31G(d,p) method was applied to optimize the molecular structures of selected Mannich bases to detect their geometrical characteristics. The DFT values (0.2214 eV and 0.2172 eV) of benzoxazine-substituted isoflavene-based Mannich bases (**9** and **10**) reflect the increase in chemical reactivity and bioactivity due to the most stable conformational arrangements. The energy gap values (0.2188 eV and 0.2181 eV) of isoflavenes with tertiary amine-based Mannich bases (**14** and **17**) reflect the increase in chemical reactivity and bioactivity due to the most stable conformational arrangements. The findings of the docking study revealed that the binding positions and binding affinities of compounds **1**, **5**, **12**, **15**, and **17** are highest against selected proteins. In conclusion, the results of the in silico docking study manifested that Mannich bases revealed the optimal ligand–receptor structure from the docked structure based on the lowest energy and number of H-bonds formed between the target and ligands. The drug-likeness and metabolite profile of the targeted drugs were confirmed by in silico ADME investigations. This study provides evidence that these selected compounds might be an important and useful target for the treatment of cancer by developing more potent drugs.

## Figures and Tables

**Table 1:** Descriptors of global reactivity calculated at the DFT level using the c(p, d) +G level for compounds **1** to **18**.

Ligand	*E*_LUMO_	*E*_HOMO_	ΔE (_HOMO_–_LUMO)_	Ionization Potential (*I*)	Electron Affinity (*A*)	Chemical Hardness (*η*)	Chemical Softness (*ζ*)	Electronegativity (*χ*)	Chemical Potential (*μ*)	Electrophilicity Index (*ω*)
**1**	−0.0365	−0.2470	0.2106	0.2470	0.0365	0.1053	4.7495	0.1418	−0.1418	0.0955
**2**	−0.0364	−0.2331	0.1968	0.2331	0.0364	0.0984	5.0826	0.1347	−0.1347	0.0923
**3**	−0.0332	−0.2168	0.1836	0.2168	0.0332	0.0918	5.4481	0.1250	−0.1250	0.0851
**4**	−0.0331	−0.2506	0.2175	0.2506	0.0331	0.1087	4.5983	0.1418	−0.1418	0.0925
**5**	−0.0360	−0.2332	0.1972	0.2332	0.0360	0.0986	5.0720	0.1346	−0.1346	0.0919
**6**	−0.0335	−0.1941	0.1607	0.1941	0.0335	0.0803	6.2243	0.1138	−0.1138	0.0806
**7**	−0.0374	−0.2160	0.1786	0.2160	0.0374	0.0893	5.6004	0.1267	−0.1267	0.0899
**8**	−0.0378	−0.2277	0.1899	0.2277	0.0378	0.0949	5.2670	0.1328	−0.1328	0.0928
**9**	−0.0340	−0.2554	0.2214	0.2554	0.0340	0.1107	4.5171	0.1447	−0.1447	0.0946
**10**	−0.0376	−0.2547	0.2172	0.2547	0.0376	0.1086	4.6047	0.1462	−0.1462	0.0984
**11**	−0.0379	−0.2291	0.1912	0.2291	0.0379	0.0956	5.2312	0.1335	−0.1335	0.0933
**12**	−0.0386	−0.1826	0.1441	0.1826	0.0386	0.0720	6.9401	0.1106	−0.1106	0.0849
**13**	−0.0382	−0.2172	0.1789	0.2172	0.0382	0.0895	5.5888	0.1277	−0.1277	0.0911
**14**	−0.0356	−0.2543	0.2188	0.2543	0.0356	0.1094	4.5714	0.1449	−0.1449	0.0960
**15**	−0.0323	−0.2109	0.1786	0.2109	0.0323	0.0893	5.6000	0.1216	−0.1216	0.0828
**16**	−0.0294	−0.1725	0.1431	0.1725	0.0294	0.0716	6.9876	0.1010	−0.1010	0.0712
**17**	−0.0372	−0.2553	0.2181	0.2553	0.0372	0.1091	4.5842	0.1462	−0.1462	0.0980

**Table 2:** The examined ligand–receptor complexes docking scores and ∆G energy of selected Mannich bases (values are expressed in kcal/mol).

Ligand	5GTY		3RHK		7JXH		6PL2	
	Docking Score	∆G Energy	Docking Score	∆G Energy	Docking Score	∆G Energy	Docking Score	∆G Energy
**1**	−2.639	−21.582	−9.893	−47.312	−8.369	−52.438	−8.159	−42.346
**2**	−3.158	−20.367	−6.533	−39.419	−5.368	−36.954	−9.41	−44.915
**3**	−3.631	−29.513	−7.456	−40.587	−6.119	−40.41	−8.346	−46.414
**4**	−3.101	−31.338	−8.082	−45.912	−5.261	−43.998	−8.798	−48.525
**5**	−3.434	−31.106	−9.242	−46.18	−6.805	−45.557	−10.655	−51.955
**6**	−3.482	−33.296	−7.627	−48.438	−6.633	−49.132	−9.343	−49.977
**7**	−3.6	−34.043	−7.044	−43.11	−8.019	−47.516	−6.088	−43.757
**8**	−3.216	−21.266	−6.932	−36.14	−4.942	−32.092	−8.756	−37.772
**9**	−2.968	−25.386	−8.072	−48.178	−7.259	−41.581	−9.399	−53.196
**10**	−3.019	−25.476	−7.298	−39.42	−6.961	−42.727	−9.051	−48.615
**11**	−3.415	−31.474	−7.375	−42.465	−6.388	−45.133	−8.175	−46.66
**12**	−4.127	−26.422	−7.163	−41.041	−7.055	−40.526	−8.883	−42.716
**13**	−3.303	−29.724	−6.464	−44.688	−5.524	−44.233	−8.583	−46.07
**14**	−3.332	−27.856	−7.602	−42.318	−5.324	−40.358	−9.083	−48.238
**15**	−3.734	−39.888	−7.207	−53.081	−8.567	−53.848	−9.447	−56.381
**16**	−3.625	−24.368	−6.725	−40.05	−5.982	−42.368	−7.891	−58.708
**17**	−4.093	−30.532	−5.953	−39.325	−5.687	−39.352	−7.22	−44.929
Erlotinib	−7.629	−54.808	−4.143	−32.362	−6.327	−52.283	−2.279	−31.124
Neratinib	−5.674	−58.645	−2.894	−36.451	−4.009	−52.789	−0.676	−28.228
Tepotinib	−9.029	−66.287	−2.811	−36.912	−7.204	−59.378	−3.339	−47.78

**Table 3:** ADME properties.

Title	mol MW	Donor HB	Accpt HB	QP logPo/w	QP logS	QPP Caco	Metab	Qplog Khsa	Human Oral Absorption	Percent Human Oral Absorption	Rule of Five	Rule of Three
1	359.424	3	3.75	3.959	−4.675	247.33	6	0.638	3	92.961	0	0
2	311.38	3	3.75	2.94	−3.605	247.961	5	0.359	3	87.013	0	0
3	367.487	3	3.75	4.408	−5.09	247.015	5	0.76	3	95.582	0	0
4	325.407	3	3.75	3.367	−4.016	262.972	5	0.439	3	89.974	0	0
5	373.451	3	3.75	4.305	−5.109	227.501	6	0.753	3	94.34	0	0
6	337.418	2	4.25	3.402	−4.218	320.009	5	0.574	3	91.702	0	0
7	434.577	2	6.25	4.176	−4.827	116.591	7	0.983	3	88.384	0	1
8	325.407	2	4.25	3.222	−3.524	320.281	5	0.424	3	90.655	0	0
9	410.555	2	6.25	3.845	−3.484	129.428	7	0.67	3	87.259	0	1
10	339.39	2	5.95	2.475	−3.198	317.19	6	0.182	3	86.203	0	0
11	379.498	1	4.25	4.955	−5.619	664.12	4	0.994	3	100	0	0
12	323.391	1	4.25	3.387	−3.927	674.659	4	0.54	3	100	0	0
13	295.337	1	4.25	2.695	−3.187	608.761	4	0.304	3	92.559	0	0
14	337.418	1	4.25	3.824	−4.387	654.841	4	0.642	3	100	0	0
15	321.375	1	4.25	3.378	−3.745	679.875	5	0.469	3	100	0	0
16	371.435	1	4.25	4.423	−4.847	722.099	5	0.836	3	100	0	0
17	325.363	2	5.95	2.253	−3.563	194.899	5	0.136	3	81.119	0	0
18	385.462	1	4.25	4.719	−5.091	720.681	5	0.926	3	100	0	0

Reference Range: Descriptors according to the QikProp user handbook; octanol/water partition coefficient was estimated using the following data: molecular weight (130.0–725.0), HBD (0.0–6.0), HBA (2.0–20.0), and QPlogPo/w. Percent Human Oral Absorption (>80% is high, 25% is bad), Human Oral Absorption (1, 2, or 3 for low, medium, or high), Rule of Five (0–4), and Rule of Three (0–3). QPPCaco (25 poor, >500 excellent).

## Data Availability

Not applicable.
